# A New Species and New Records for the Subfamily Aleocharinae from Turkey

**DOI:** 10.1673/031.013.14001

**Published:** 2013-11-30

**Authors:** Osman Sert, Yavuz Turan, Senem Fırat, Burcu Şabanoğlu

**Affiliations:** University of Hacettepe, Faculty of Science, Biology Department, Applied Biology Section, 06800, Beytepe, Ankara, Turkey

**Keywords:** genitalia, *Gyrophaena*

## Abstract

In this study, a new Aleocharinae (Coleoptera: Staphylinidae) species from Turkey, *Gyrophaena cagatay* sp. n. (Aksaray), is described and illustrated. In addition, new records are presented for seven species: *Atheta hygrotopora* (Kraatz, 1856)*, Atheta incognita* (Sharp, 1869)*, Atheta ripicola* Hanssen, 1932*, Brachyusa concolor* Erichson, 1839, *Ischnopoda leucopus* (Marsham, 1802), *Ischnopoda subaenea* Eppelsheim, 1890, and *Liogluta granigera* Kiesenwetter, 1850. Photographs of the habitus of male and female specimens are presented. The 8^th^ tergite and aedeagus of the male and the spermatheca of the female are also illustrated for the new species. Differential diagnosis guidelines are given for comparisons with *Gyrophaena rousi* Dvořak, 1966.

## Introduction

The members of *Gyrophaena*, Aleocharinae (Coleoptera: Staphylinidae), are obligate inhabitants of fresh mushrooms as larvae and adults. They live on both polypore and gilled mushrooms. Adults appear on mushrooms soon after the gills are exposed or the hymenium area becomes active, and both larvae and adults occupy more mature mushrooms. Gyrophaenines inhabit only fresh mushrooms and are usually among the first insects to appear on them (Ashe 1984). Until recently, the genus *Gyrophaena* Mannerheim, 1830 was represented by 10 species in Turkey (Anlaş 2009). Following Assing's recent studies (2009 and 2011), two new species and three new records were added to this number. With the newly described species, the total number of *Gyrophaena* species found in Turkey has been raised to 16.

## Materials and Methods

Field studies were conducted from 2009–2011 in 13 cities in the Central Anatolian Region of Turkey. Insects were sampled by using aerial nets, sifting through ground and leaf debris, inspecting mushrooms and using burying traps. Insects were killed with 95% ethanol and ethylacetate. The coordinates of the localities of the specimens were recorded using a GPS. At the end of the field study, the insects were brought to the laboratory for identification and placed in a collection. The identifications were made using the methods from Lohse (1974), Pasnik (2006), Brundin (1944), and Strand and Vik (1964). The province, county, coordinates of localities, altitude, and collecting dates, together with the numbers of male and female specimens, are given below.

Genitalia were extracted using standard methods. Photographs of the habitus, male 8^th^ tergite, aedeagus, and female spermatheca were taken using a Leica MZ 16A stereoscopic microscope (Leica Microsystems, http://www.leica-microsystems.com). *Gyrophaena cagatay* sp. n. was compared to the most similar species, *Gyrophaena rousi* Dvořak, by means of the male 8^th^ tergite and the structure of the aedeagus.

## Results and Discussion

**Figure 1.**
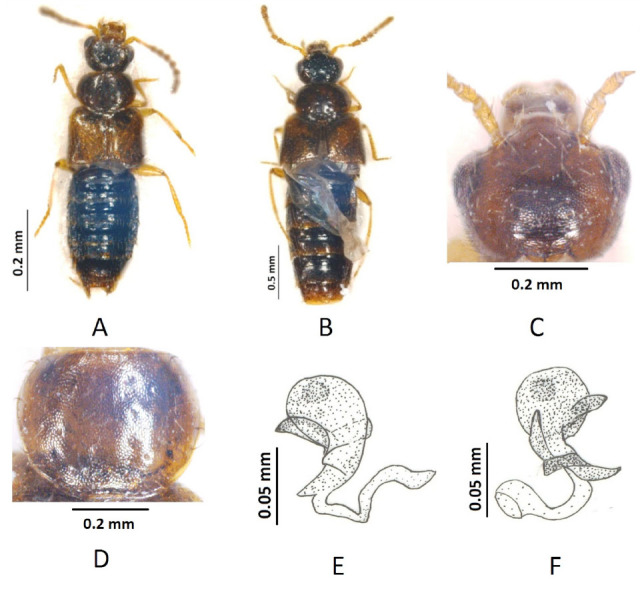
*Gyrophaena cagatay* sp. n. A: male habitus; B: female habitus; C: head; D: pronotum; E and F: spermatheca. High quality figures are available online.

**Figure 2.**
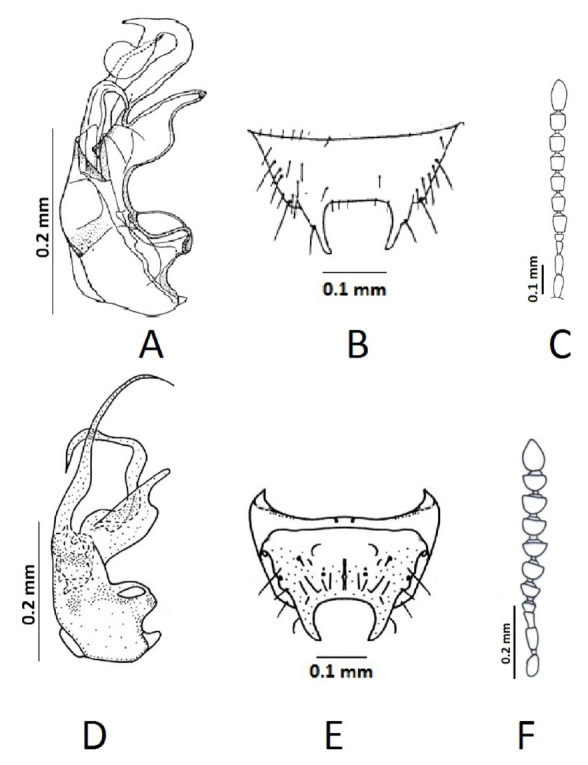
(A–C) *Gyrophaena rousi* Dvořak, 1966, (from Welch, 2000); (D–F) *Gyrophaena cagatay* sp. n.; A and D: the structure of the median lobe of the aedeagus (lateral view); B and E: male 8th tergite; C and F: antennae. High quality figures are available online.

**Figure 3.**
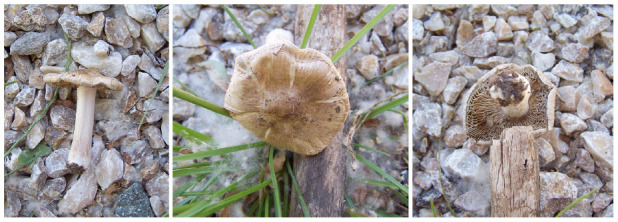
The mushroom from which *Gyrophaena cagatay* sp. n. specimens were sifted. High quality figures are available online.

***Gyrophaena cagatay* sp. n.**
**Type Locality:** Holotype, 1 ♂ Aksaray province, Central county, Helvadere village, Hasan Mountain, 38°09′55″ N, 34°12′27″ E, 1708 m a.s.l., 01.VII.2011 leg: Y. Turan; Paratype, 5 ♂♂, 3 ♀♀, same data as holotype, (Specimens are deposited in our private collection.), sifted from mushroom.
**Description:** Habitus as in Figure 1A and B. Total body length from anterior margin of labrum to posterior margin of 8^th^ tergite is 2–2.3 mm. Head dark brown or blackish, glossy; pronotum dark brown, with basal and lateral margins pale brown; elytra brown or yellowish- brown with posterior angles more or less darkened; abdomen black or dark brown, posterior margin of each tergite paler; mouthparts, legs and 1^st^–4^th^ segments of antennae yellowish, 5^th^—11^th^ segments brown.
  Head distinctly transverse, 1.26–1.3 times wider than its length, head length from anterior margin of clypeus to neck is 0.27 mm and maximal head width including eyes is 0.34 mm, vertex surface with round, distinct, and sparse punctures (Figure 1C); length of antennae is 0.68 mm, antennae moderately long, segment II narrower and shorter than segment I, segment III narrower and shorter than segment II, segment IV short, distinctly widened apically, segments V–X approximately equal in length and width, slightly widened apically.
  Pronotal length along midline is 0.58 mm, pronotum distinctly transverse, 1.4–1.5 times wider than its length and 1.5–1.6 times wider than head; surface with rounded, few, and sparse punctures, wider at medial (Figure 1D). Maximal elytral length from base to posterior margin is 0.54 mm and total width of elytra is 0.71 mm, elytra 1.3–1.4 times wider than pronotum, surface with sparse and small punctures. Abdomen short, more or less widened. Male: 8^th^ tergite with two small and two slightly wider, rounded punctures medially; posterior margin of 8^th^ tergite without short appendages and slightly concave, laterally on each side with two long, pointed and slightly inward curved process (Figure 2E). Entire body with dense and distinct reticulate microsculpture. Aedeagus bulbus with a proximal tooth-like process; median lobe with ventral process wide at base, slightly convex on ventral side and finger-shaped narrowed through apical; dorsal inner sac sclerite narrow and whip-shaped with pointed apex; ventral inner sac sclerite sinuous and sickleshaped with pointed apex (Figure 2D). Spermatheca, with wide head; neck with a collarshaped projection on one side and tuber-like projection on the other side; body with a pointed apex (Figure 1E and F).
**Etymology:** The new species is named after the our honorable advisor, Professor Neşe Çağatay, PhD.
**Distribution and Bionomics:** The specimens were sifted from mushrooms (Figure 3) from the Hasan Mountain hillside, Aksaray province at an altitude of 1708 m a.s.l.
**Comparative Notes:**
  ***Gyrophaena* sp. n.** undoubtedly most closely resembles *Gyrophaena rousi* Dvořak, 1966. The antennae of *Gyrophaena cagatay* sp. n, especially the second segment, is thicker than that of *G. rousi* in general. The third segment is slightly longer and thinner than in *G. rousi*. Segments V–X are apparently transverse, broader than long. The last segment is narrowed through apical and has a length:width ratio of 1.6:1 instead of the 2.1:1 ratio in *G. rousi* from Welch, 2000 (Figure 2 C and F). New species could be reliably distinguished from congeners by means of the 8^th^ male tergite and the aedeagus (Figure 2 A, B, D, and E). The new species' 8^th^ male tergite and aedeagus are given for comparison. The aedeagus differs from that of *G. rousi*, which is the most similar species, in the male genitalia structure. *Gyrophaena cagatay* sp. n. could be differentiated based on the following characteristics: the widened part of the median lobe is narrower than that of *G. rousi*; the dorsal inner sac sclerite is approximately twice as long as the median lobe and distinctly curved inward through apical, while it is approximately equal in length to the median lobe and slightly curved inward in *G. rousi*; the ventral inner sac sclerite is sinuous and sickle shaped with two foldings on the medial and distal parts, while it is stick-shaped and has only one folding on the distal part in *G. rousi*.

### New Records

**Subfamily Aleocharinae Fleming, 1821 Tribe: Athetini Casey, 1910 *Atheta hygrotopora* (Kraatz, 1856)**
**Material examined:** Niğde-Bor, 1304 m a.s.l., 37°49′39″ N, 34°41′19″ E, 19.VIII.2009, 2 ♂♂; Kırşehir-Çiçekdağı, 888 m a.s.l., 39°42′07″ N, 34°08′43″ E, 26.V.2010, 1 ♂; Niğde-Çiftlik, 1560 m a.s.l., 38°10′14″ N, 34°29′15″ E, 03.VI.2010, 1 ♂; NiğdeÇamardı, 1486 m a.s.l., 37°49′55″ N, 34°58′56″ E, 18.VIII.2010, 8 ♂♂, 4 ♀♀; Niğde-Çamardı, 1355 m a.s.l., 37°45′04″ N, 35°00′13″ E, 18.VIII.2010, 1 ♂; Kayseri- Yeşilhisar, 1317 m a.s.l., 38°20′11″ N, 34°59′06″ E, 19.VIII.2010, 5 ♂♂; NiğdeÇamardı, 1486 m a.s.l., 37°49′55″ N, 34°58′56″ E, 21.VIII.2010, 6 ♂♂ , 2 ♀♀; Niğde-Çamardı, 1479 m a.s.l., 37°49′56″ N, 34°58′57″ E, 29.VI.2011, 5 ♂♂, 3 ♀♀, det: S. Fırat, confirmed by Volker Assing.
**Distribution:** According to Smetana, 2004, *A. hygrotopora* was known to exist in Austria, Belgium, Bosnia Herzegovina, Bulgaria, Czech Republic, Denmark, Estonia, Finland, France, Great Britain, Germany, Greece, Ireland, Italy, Luxembourg, The Netherlands, Norway, Poland, Romania, Slovakia, Russia (South European Territory), Sweden, Switzerland, Ukraine, and Kazakhistan. The species is reported to exist in Turkey for the first time. The specimens were collected from river banks by sifting sand.

***Atheta incognita* (Sharp, 1869)**
**Material examined:** Nevşehir-Kozaklı, 1018 m a.s.l., 39°20′49″ N, 34°39′10″ E, 25.V.2010, 1 ♂, 1 ♀, det: Y. Turan
**Distribution:** According to Smetana, 2004, *A. incognita* was known to exist in Austria, Belgium, Bulgaria, Russia (Central European Territory), Czech Republic, Denmark, Estonia, Finland, France, Great Britain, Germany, Hungary, Ireland, Italy, The Netherlands, Norway, Russia (North European Territory), Poland, Romania, Slovakia, Slovenia, Sweden, and Switzerland. The species is reported to exist in Turkey for the first time. The specimens were collected from river banks.

***Atheta ripicola* Hanssen, 1932**
**Material examined:** Niğde-Merkez, 1596 m a.s.l., 38°00′01″ N, 34°51′32″ E, 30.IV.2011, 2 ♂♂, det: S. Fırat.
**Distribution:** According to Smetana, 2004, *A. ripicola* was known to exist in Austria, Denmark, Estonia, Finland, France, Germany, Italy, Latvia, Russia (North European Territory), Norway, Poland, Sweden, Switzerland, Russia (East and West Siberia), and Mongolia. The species is reported to exist in Turkey for the first time. The specimens were collected from a reed field.

***Liogluta granigera* Kiesenwetter, 1850**
**Material examined:** Ankara-Kızılcahamam, 1752 m a.s.l., 40°40′14″ N, 32°45′28″ E, 21.X.2011, 1 ♀, det: Y. Turan.
**Distribution and Bionomics:** According to Smetana, 2004, *L. granigera* was known from Austria, Belgium, Bosnia Herzegovina, Bulgaria, Belarus, Croatia, Russia (Central European Territory), Czech Republic, Denmark, Estonia, Finland, France, Great Britain (including the Channel Islands), Germany, Georgia, Hungary, Italy, (including Sardegna, Sicilia, and San Marino), Lithuania, The Netherlands, Norway, Russia (North European Territory), Poland, Portugal, Slovakia, Russia (South European Territory), Sweden, Switzerland, Yugoslavia (Serbia and Montenegro), East and West Siberia, and North Korea. The species is reported to exist in Turkey for the first time. The specimen was collected by sifting mushrooms in a forest.

**Tribe: Oxypodini Thomson, 1859 *Brachyusa concolor* (Erichson, 1839)**
**Material examined:** Konya-Seydişehir, 1610 m a.s.l., 37°32′50″ N, 32°09′15″ E, 02.VI.2009, 2 ♂♂; Aksaray-Güzelyurt, 1127 m a.s.l., 38°15′53″K, 34°17′25″D, 23.V.2010, 1 ♀, det: S. Fırat.
**Distribution:** According to Smetana, 2004, *B. concolor* was known to exist in Austria, Belgium, Russia (Central European Territory), Czech Republic, Denmark, Finland, France, Great Britain (including the Channel Islands), Germany, Georgia, Hungary, Latvia, Lithuania, The Netherlands, Norway, Russia (North European Territory), Poland, Romania, Slovakia, Sweden, Ukraine, and East Siberia. The species is reported to exist in Turkey for the first time. The specimens were collected from river banks.

**Tribe: Tachyusini Thomson, 1859 *Ischnopoda leucopus* (Marsham, 1802)**
**Material examined:** Sivas-Hafik, 1353 m a.s.l., 39°57′53″ N, 37°22′33″ E, 07.VII.2010, 1 ♂, 1 ♀, det: Y. Turan.
**Distribution:** According to Smetana, 2004, *I. leucopus* was known to exist in Austria, Belgium, Russia (Central European Territory), Czech Republic, Denmark, Estonia, Finland, France, Great Britain (including the Channel Islands), Germany, Ireland, Italy, (including Sardegna, Sicilia, and San Marino), Lithuania, The Netherlands, Norway, Russia (North European Territory), Poland, Slovakia, Sweden, Switzerland, Ukraine, and East and West Siberia. The species is reported to exist in Turkey for the first time. The specimens were collected from river banks under stones.

***Ischnopoda subaenea* Eppelsheim, 1890**
**Material examined:** Sivas-Şarkışla, 1456 m a.s.l., 39°16′19″ N, 36°34′59″ E, 08.VII.2010, 1 ♂, det: Y. Turan, confirmed by Grzegorz Paśnik.
**Distribution:** According to Smetana, 2004, *I. subaenea* was reported to exist only in Georgia. The species is reported to exist in Turkey for the first time. The specimen was collected from a river bank under a stone.

## References

[bibr01] Anlaş S. (2009). Distributional checklist of the Staphylinidae (Coleoptera) of Turkey, with new and additional records.. *Linzer Biologische Beiträge*.

[bibr02] Ashe JS. (1984). Generic revision of the subtribe Gyrophaenina (Coleoptera: Staphylinidae: Aleocharinae) with a review of the described subgenera and major features of evolution.. *Quaestiones Entomologicae*.

[bibr03] Assing V. (2009). On the Staphylinidae of Turkey. VI. Thirteen new species and additional records (Coleoptera).. *Koleopterologische Rundschau*.

[bibr04] Assing V. (2011). On the Staphylinidae of Turkey VIII. Eleven new species, two new synonymies, a new combination, and additional records (Coleoptera: Staphylinidae).. *Koleopterologische Rundschau*.

[bibr05] Brundin L. (1944). Monographie der palaearktischen Arten der Atheta- Untergattung Hygroecia (Coleoptera, Staphylinidae).. *Annalen des Naturhistorischen Museums in Wien*.

[bibr06] Lohse GA., Freude H, Harde KW, Lohse GA (1974). Family Staphylinidae.. *II. Die Kafer Mitteleuropas.*.

[bibr07] Paśnik G. (2006). Taxonomy and phylogeny of the World species of the genus *Ischnopoda* STEPHENS, 1837 (Coleoptera, Staphylinidae, Aleocharinae).. *Zootaxa*.

[bibr08] Smetana A., Löbl I, Smetana A (2004). Family Staphylinidae (except subfamilies Pselaphinae and Scaphidiinae).. *Catalogue of Palaearctic Coleoptera. Volume 2. Hydrophiloidea, Histeroidea, Staphylinoidea*..

[bibr09] Strand A, Vik A. (1964). Die Genitalorgane der nordischen Arten der Gattung Atheta Thoms. (Col., Staphylinidae).. *Norsk Entomologisk Tidsskrift*.

[bibr10] Welch RC. (2000). Gyrophaena rousi Dvroak 1966 (Col., Staphylinidae) New to Britain.. *Entomologist's Monthly Magazine*.

